# Effects of Acute and Chronic Exercise on Immunological Parameters in the Elderly Aged: Can Physical Activity Counteract the Effects of Aging?

**DOI:** 10.3389/fimmu.2018.02187

**Published:** 2018-10-10

**Authors:** Maha Sellami, Maha Gasmi, Joshua Denham, Lawrence D. Hayes, Dan Stratton, Johnny Padulo, Nicola Bragazzi

**Affiliations:** ^1^Sport Science Program (SSP), College of Arts and Sciences (QU-CAS), University of Qatar, Doha, Qatar; ^2^Higher Institute of Sport and Physical Education of Ksar Said, Mannouba, Tunisia; ^3^School of Health and Biomedical Sciences, RMIT University, Melbourne, VIC, Australia; ^4^Active Ageing Research Group, Department of Medical and Sport Sciences, University of Cumbria, Lancaster, United Kingdom; ^5^Cellular and Molecular Immunology Research Center, London Metropolitan University, London, United Kingdom; ^6^University eCampus, Novedrate, Italy; ^7^Department of Health Sciences (DISSAL), Postgraduate School of Public Health, University of Genoa, Genoa, Italy

**Keywords:** physical activity, age, immunosenescence, innate immune system, adaptive immune system

## Abstract

Immunosenescence is characterized by deterioration of the immune system caused by aging which induces changes to innate and adaptive immunity. Immunosenescence affects function and phenotype of immune cells, such as expression and function of receptors for immune cells which contributes to loss of immune function (chemotaxis, intracellular killing). Moreover, these alterations decrease the response to pathogens, which leads to several age-related diseases including cardiovascular disease, Alzheimer's disease, and diabetes in older individuals. Furthermore, increased risk of autoimmune disease and chronic infection is increased with an aging immune system, which is characterized by a pro-inflammatory environment, ultimately leading to accelerated biological aging. During the last century, sedentarism rose dramatically, with a concomitant increase in certain type of cancers (such as breast cancer, colon, or prostate cancer), and autoimmune disease. Numerous studies on physical activity and immunity, with focus on special populations (i.e., people with diabetes, HIV patients) demonstrate that chronic exercise enhances immunity. However, the majority of previous work has focused on either a pathological population or healthy young adults whilst research in elderly populations is scarce. Research conducted to date has primarily focused on aerobic and resistance exercise training and its effect on immunity. This review focuses on the potential for exercise training to affect the aging immune system. The concept is that some lifestyle strategies such as high-intensity exercise training may prevent disease through the attenuation of immunosenescence. In this context, we take a top-down approach and review the effect of exercise and training on immunological parameters in elderly at rest and during exercise in humans, and how they respond to different modes of training. We highlight the impact of these different exercise modes on immunological parameters, such as cytokine and lymphocyte concentration in elderly individuals.

## Aging and its impact on the immune system

Immunosenescence or immunopause is a complex, multi-factorial aging-related phenomenon characterized by a series of biological events, including (a) alteration of immune function, quantified by a reduction in humoral and cellular immunity, (b) increase in the inflammatory and oxidation background (inflammaging and oxi-inflammaging), and (c) production and release of auto-antibodies leading to the insurgence of autoimmune disorders, as briefly overviewed in Table 1 (1).

**Table 1:** Aging-related immunosenescence or immunopause.

**Phenomenon/event**	**Details**
Reduction in immune response	▪ Thymus involution and atrophy and reduction of primary lymphopoiesis ▪ Decline in T- and B-cells, especially in naïve T- and B-cells ▪ Decrease in antibody production and delay in antibody response to new stimuli and antigens ▪ Decline in hematopoietic stem cells and progenitor cell function ▪ Decrease in lymphoid potential and of lymphoid progenitors (T-cell progenitors) ▪ Decrease in the number of dendritic cells and their ability to recognize pathogens
Increase in the inflammatory and oxidation background (inflammaging and oxi-inflammaging)	▪ Increased secretion of pro-inflammatory cytokines released by M1–pro-inflammatory macrophages and other cells ▪ Accelerated tissue damage
Production and release of auto-antibodies	▪ Increased autoimmune manifestations

Deterioration of the immune system with age is mostly due to biological factors such as genetics and interactions with environmental factors (like exposure to infectious agents, including CMV or cytomegalovirus) imposing metabolic alterations caused by unhealthy lifestyles (poor exercise, inadequate diet) and prolonged physiological stress (2, 3).

Aging primarily influences immunity through changes in thymus structure and activity (i.e., thymus atrophy) and reduction of primary lymphopoiesis (4, 5). Additionally, aging is associated with a decline in naïve T-cells, accumulation of memory T-cells, and a decrease in antibody production (6–8). Aging is also associated with a decline in hematopoietic stem cells (HSCs) and progenitor cell function, which results in increased production of myeloid lineage cells and a decrease in lymphoid potential (9). Thus, the quality and the number of lymphoid progenitor cells reduce with age and the cellular immune compartment becomes skewed toward a myeloid lineage (10, 11).

Moreover, aging perturbs the inflammatory state by increasing secretion of pro-inflammatory cytokines (i.e., interleukin-1 [IL-1], tumor necrosis factor alpha (TNF-α), interleukin-6 [IL-6], and C reactive protein [CRP]) (12). In fact, with advanced age, macrophages become more pro-inflammatory releasing higher amounts of TNF-α and interleukin-12 (IL-12) (13), which can accelerate tissue damage (14). The perturbed secretory state of senescent cells is known as the senescence-associated secretory phenotype and contributes to the aging process (15–17).

A further major aging-related event also occurs in the form of increased production and release of auto-antibodies, leading to a higher number of autoimmune events and manifestations among the elderly (1, 18).

## Physical activity as a tool to counteract immunosenescence

Importantly, some lifestyle interventions can preserve the normal course of aging and, ultimately, prevent premature immunosenescence. Among these interventions, diet and exercise training (multiple single bout of exercise) are the most studied non-pharmacological strategies to fight the age-associated decline in immunity (19–21).

In fact, exercise training has been shown to induce transient changes in immunity responses at rest and in response to efforts (i.e., recovery following efforts). Exercise training or the “chronic exercise” intervention can be defined as a repeated amount of bouts of exercise during a short or long-term period of time) while, the “acute exercise” can be defined as a single bout of exercise.

The available scholarly literature seems to suggest that chronic exercise is a safe mode of intervention to prevent immunosenescence, chronic low-grade inflammation and improve the effectiveness of flu vaccination in elderly populations without harmful side effects (22–24).

Other recent studies have indeed suggested that chronic exercise exerts a positive effect on cardiovascular health (25–27) as well as on the immune system (28–31).

On the other hand, it has been well demonstrated that physiological responses to acute and long-term adaptations of immunity to exercise are dependent on exercise type or dose (low intensity (<40% VO_2max_), moderate (40-69%VO_2max_) vigorous (70-90%VO_2max_), or very high intensity (>90% VO_2max_)). Following an overly intense workout, some authors have interpreted measurements to show that there is a general decrease in immunity for several hours after exercise, termed the “open-window theory of susceptibility to infections,” showing, on the contrary, that, chronic exercise can enhance immunity rather than suppress immune competency among athletes (32, 33). An array of parameters including fatigue, nutritional deficiency, psychological stress, or environmental exposures and not just exercise *per se* can, indeed, explain the apparently higher rate of upper respiratory tract infections (URTIs) in athletes compared to general population (32, 34, 35). Exercise training can be considered as a kind of “immunotherapy,” potentially representing a highly cost-effective measure that can dramatically improve human quality of life (36).

As for acute exercise, the changes in immunity response seems to be altered by exercise type and form (i.e., endurance, resistance or sprint training). For example, the endurance training which refers to regular exercises at low to moderate intensity, is generally seen to enhance the aerobic system and cardio-respiratory function together with the exercised muscles. This type of training is essential for sports like running a marathon, swimming a long distance, or climbing mountains, which have recently become more practiced among elderly subjects. The resistance training includes all forms of exercise that forces skeletal muscles (not the involuntary muscles) to contract in response to some type of force that “resists” to the movement with or without equipment (i.e., weight training, isometric exercise, weight machines…etc.). It is commonly used to increase muscular strength and may reduce metabolic, cardiovascular disease, and risk of fall in 70–75 year old subjects (37, 38).

Finally, sprint training which is considered the most intense training mode because it includes short bouts of running exercise at high speed (i.e., race over short distances such as 10, 100, 800 m). Benefits of sprinting for the middle aged (40–50 years) including building muscles, burning fat, relieving stress and also improvement of the endocrine system (39–41) have been well demonstrated, however, few and unclear data were reported in elderly subjects with regard to all the body's system and especially to the immunity response, which was more apparent in other type of training.

One of the major concerns in training adaptation in the immune system concerns changes in catecholamines, with a blunted neuro-endocrine response and adrenergeric receptors being down-regulated. In fact, previous studies have demonstrated that chronic exercise such as sprint and resistance training may counteract the negative effect of age on catecholamines and growth hormones in 40 year-old men (40–42). As catecholamines modulate immune cell function (43, 44), it is therefore important to highlight the impact of these type of exercises training on immunity in the elderly population.

In the subsequent sections, building on an extensive search of the literature based on important discoveries of Nieman and collaborators from the 90's (45–49) to recent data of 2018, we will discuss the current understanding of different modes of exercise training on immunological parameters and mechanisms that are implicated in immunosenescence in older subjects. As there is little research in this area, we have also mentioned in some parts that only studies in young subjects (20–30 years) were conducted. In fact, in the literature, there is a lack of consensus concerning the definition of elderly/older subject: according to the World Health Organization (WHO), this category includes individuals aged >60 or 65 years old, whereas the oldest subject is a person aged >80 years old.

## Impact of acute exercise on immune cells

A single bout of exercise is known to stimulate immune cells during efforts and during recovery. Evidence indicates that mechanisms underlying exercise associated with immune function alteration are related to several factors such as neuro-endocrine system stimulations (catecholamines, cortisol), and metabolic (i.e., carbohydrate, antioxidants, or prostaglandin) (50, 51) as well as to cardiac output, blood flow, blood pressure, and shear forces, among others.

Acute exercise impacts on circulation and leaves blood to travel to tissues where they are more likely to encounter infected cells or body cells that have become cancerous. Some studies seem to suggest that the acute effects of exercise (e.g., apoptosis of some cells) can stimulate a mobilization of hematopoietic stem cells from bone marrow and of senescent immune cells from the peripheral tissues to the circulation (22, 52–55). This could justify and explain why in older subjects, in which these mechanisms are impaired by aging, response to acute exercise is different from younger individuals, as shown by some studies reported in Table 2.

**Table 2:** The effect of acute exercise on the immune system.

**Reference**	**Subjects' age and sex**	**Type of acute exercise**	**Time of samples collection**	**Results**
Crist et al. (56)	Sex: female Age:72 ± 1 years Number: 14 (7 allocated to a 16-weeks program of physical exercise training vs. 7 controls)	Acute treadmill exercise	Samples collected 20 min before and after treadmill exercise	↑ NK cells activity (specific lysis 38.2 vs. 28.8% in controls, *p* < 0.05 after experimental period; specific lysis 38.2–57.4 %, vs. 28.8–37.8% in controls, *p* < 0.05)
Fiatarone et al. (57)	Sex: female Age: 65 years and above vs. 21-39 years Number: 9 vs. 8	Cycling ergometer test	Samples collected 15 min before the exercise, during the peak of the exercise and 15 min after the exercise (recovery period)	↑ NK activity and response
Cannon et al. (58)	Sex: male Age: 55–74 years vs. 22–29 years Number: 21 randomly allocated (double-blind placebo-controlled trial)	Running on an inclined treadmill (intense eccentric exercise) at an intensity of 75% of maximal heart rate	Samples collected 1 day before, the day of the test, 1, 2, 5, and 12 days after the test	↑circulating neutrophils in young vs. old subjects. ↑intramuscular CK in young vs. old subjects. Dietary supplementation with vitamin E eliminated the differences between young and old subjects.
Shinkai et al. (59)	Sex: male Age Number: 21	Exercise on a cycle ergometer for 60 min at 60% of VO_2max_	Samples collected every 30 min during the text and after 120 min, during the recovery period	↑CD3+, CD19+, CD4+, CD8+, and CD16+ cells during exercise ↓ CD3+, CD19+, CD4+, CD8+, and CD16+ cells 30 min after exercise
Cannon et al. (60)	Sex: NR Age: 61–72 vs. 20-32 Number: 12 vs. 9	Eccentric exercise (45 min at 78 % of maximum heart rate)	Samples collected immediately after exercise	↑plasma des-Arg-C3a (increase of 21%) ↑circulating neutrophils (increase of 66 ± 10% in 4–6 h) ↑CK (increase of 135 ± 25% in 24 h) Increases were smaller in older vs. younger subjects.
Mazzeo et al. (61)	Sex: male Age: 69 ± 5 vs. 26 ± 3 Number: 9 vs. 6	Bicycle ergometer (submaximal exercise at 50% peak work capacity) for 20 min	Samples collected at rest and immediately after exercise	↑lymphocyte population (both CD4+ and CD8+ subsets) ↑ lymphocyte proliferation
Bruunsgaard et al. (62)	Sex: male and female Age: 76–80 (median 78) vs. 19–31 (median 23) Number: 10 vs. 10	Maximal bicycle exercise (for 17–20 min)	Samples collected prior to the exercise (after 15 min of rest) and in the min after peak exercise	Redistribution of previously activated cells with an increased replicative story
Ceddia et al. (63)	Sex: male and female Age: 65.3 ± 0.8 vs. 22.4 ± 0.7 Number: 33 vs. 14	Acute maximal exercise (modified Balke treadmill exercise) with speed set at 2.5–3.0 and 3.5–4.0 mph for the old and young subjects, incrementing in 2-min stages with a 2% increase in grade at each stage	Samples collected immediately before, after and 20 min post-exercise	↑CD4+ cells ↑CD8+ cells ↑neutrophils ↑lymphocyte proliferation
Colbert et al. (64)	Sex: male and female Age: 70–79 Number: 3075	Moderate weekly physical activity (at least 180 min/week of walking, occupational/volunteer physical activities)	Self-administered questionnaire	↓CRP ↓IL-6 ↓TNF-alpha concentrations
Hamada et al. (65)	Sex: male Age: 66–78 vs. 23-35 Number:15 vs. 15	Acute eccentric exercise (45 min of downhill running, 16% descent, at 75% VO_2max_)	Samples collected 24 h before and 72 h after exercise	↑TNF-α mRNA ↑IL-1mRNA ↔IL-6 mRNA ↑CD18 ↑TGF-β1
Stewart et al. (66)	Sex: male and female Age: 71 vs. 25 Number: 60	12 weeks (3 days/week) of endurance (20 min) and resistance exercise (eight exercises, two sets)	Samples collected at rest, before and after training	↓IL6 ↓TLR4 ↔TLR2 ↔IL-1beta ↔TNF-alpha
McFarlin et al. (67)	Sex: male and female Age: 60–80 Number: 84	Modified Balke submaximal treadmill test	Samples collected 30 min after seated rest in a quiet room	↑hsCRP
Ludlow et al. (68)	Sex: male and female Age: 60.3 ± 4.9 (50–70) Number: 69	Estimated physical activity and energy expenditure	Validated questionnaire	↑ leukocyte telomere length in moderate exercisers compared to low and high exercisers. Association between telomerase activity and hTERT genotype with the TT genotype ↔leukocyte telomerase activity
Puterman et al. (69)	Sex: female Age: 54-82 Number: 63	Vigorous activity reported in daily diminutes (ranging from 0 to 53) for three consecutive days	Self-reported physical activity	Perceived stress unrelated to telomere length
Spielmann et al. (70)	Sex: male Age: 18–61 Number: 102	Cycling ergometer test	Samples collected after a 5-min period of seated rest	↑proportion of naïve ((KLRG1-/CD28+)) CD8+ T-cells ↓proportions of senescent/exhausted (KLRG1+/CD57+; KLRG1+/CD28-) CD4+ and CD8+ T-cells
Spielmann et al. (71)	Sex: male Age: 50–64 vs. 20–34 Number: 16 vs. 16	Sub-maximal cycling test (30-min at ~80–85% peak cycling power)	Samples collected after 5 min of seated rest, immediately after and 1 h after exercise cessation	Redeployment of CD8+ T cells and KLRG1+/CD28- and CD45RA+/CCR7- CD8+ subsets
Bigley et al. (72)	Sex: male Age: 50–64 vs. 23–39 Number: 40	Cycling exercise for 30 min at 80% of maximum power	Samples collected immediately after the exercise and after 1 h	Redeployment of NK-cells
Bartlett et al. (73)	Sex: male and female Age: 67 ± 5 (for male) and 66 ± 5 (for female) for cases vs. 23 ± 4 for controls Number: 211 vs. 10 healthy young controls	Different levels of physical activity	Accelerometry wear (measurements taken for a week)	↔IL-6 ↔IL-8 ↔MCP-1 ↔CRP ↔IL-10 ↔IL-13 ↑CD11b ↑neutrophil ↑migratory dynamics toward IL-8 ↔CXCR1 ↔CXCR2
Silva et al. (74)	Sex: male Age: 65–85 Number: 46	Untrained vs. moderate and intense training lifestyle	Self-administered questionnaires	↓CD45RA+ CCR7- CD4+ and CD8+ T-cells ↑central memory CD4+ T-cells ↑effect memory CD8+ T-cells ↔CD28- T-cells ↑telomere length in CD4+ and CD8+ T-cells
Minuzzi et al. (75)	Sex: male and female Age: 53.5 ± 8.94 years Number: 19 vs. 10	Cycle ergometer test	Samples collected before, and after 10 min and 1 h	↓percentage of senescent naïve, central memory and effector memory CD8+ T-cells and senescent naïve and effector memory CD4+ T-cells ↑SLEC CD8+ T-cells ↓naïve CD8+ T-cells.

*↑, greater response; ↓, lower response; ↔, No difference due to exercise training; CD, cluster of differentiation; URTI, upper respiratory tract infection; CK, creatine kinase; CRP, C-reactive protein; IL: interleukin; IFN: interferon;$\dot{\;V}$O_2peak:_peak oxygen uptake; NK, natural killer; NR, not reported*.

Furthermore, noradrenaline is responsible for the effects of acute exercise on lymphocyte changes, including natural killer (NK)-cell and T-cell activity (76). Increases in catecholamine with growth hormones mediate the changes in neutrophils levels and control lymphopenia and neutrocytosis during long duration exercise. In addition, glutamine, the abundant amino acid found in muscles, is known to stimulate *in vitro* lymphocyte proliferation, lymphokine activated killer cell action, and cytokine release (77). During intense exercise, blood, and muscle levels of glutamine and glucose tend to fall, which may explain how a possible “immunosuppression” is likely to occur, despite existing data that contradict this relationship between glutamine and immunosuppression (77). According to some authors, exercise-induced apoptosis is a necessary process that accelerates the removal of damaged cells without inducing a pronounced inflammatory status, which may, instead, enhance body function (78).

The magnitude of changes in immune cell levels during acute exercise and if it is dependent on exercise intensity, is still debated (22, 79–81) and most previous work investigated the effect of exercise on immunity generally in young subjects (20–30 years) while no study or very few investigations focused on the elderly population.

The proliferation of lymphocytes is considered to correlate with the response of the adaptive immune system to training. Studies use several types of mitogens to stimulate lymphocytes such as concanavalinA (con-A), poke weed mitogen (PWM), phytohaemagglutinin (PHA). Other studies, have used interleukin-2 (IL-2), purified derivative of tuberculin (PPD), and lipopolysaccharide to induce the proliferation of lymphocytes. The data are, however, equivocal. For instance, Espersen et al. (82) have shown that proliferation of lymphocytes to mitogens (such as, PHA, con A, PWM) increased 2 h after exercise. In contrast, other studies indicate that lymphocyte response to PHA, con-A and especially to PPD was depressed after 2.5–3 h of heavy exercise (a complete 42, 195-km marathon) in a sample of 4 male subjects aged 25–50 years old compared with eight highly conditioned long-distance runners and 59 controls immunized with tetanus toxoid vaccine (83). Samples were collected at 30 min before, 30 min, 3 h and 1 day after the completion of the run. However, despite the depression of lymphocyte transformation response, no changes in lymphocyte count were observed (from 3,196 to 2,451 cells per mm^3^) and no effects on antibody-forming capacity could be detected. This lymphocyte transformation response was transient (24-h recovery period). Granulocytosis, increased plasma cortisol (from 0.48 to 1.08 μmol/l) and leucocytosis (from 7,600 to 19,609 cells per mm^3^) were also found.

In order to demonstrate the effects of age and mode of exercise (in terms of intensity and duration), the impact of single exercise bouts on changes in leukocyte subsets and other immunological parameters is summarized in Table 2.

It should be emphasized that assessing cell functions in response to acute exercise may be subject to different confounding factors (such as CMV serostatus), unless is assessed on a per cell and per phenotype basis (32, 84).

In the next paragraphs, we will introduce different cell types and we will briefly explain how they respond to exercise.

**Monocytes**, which represent 2–12% of circulating leukocytes, can differentiate into macrophages and myeloid lineage dendritic cells (DCs). Aging is responsible for the pro-inflammatory phenotype of these cells such as expression of the Cluster of differentiation 16 (CD16) and increased levels of TNF-α, IL-6, and the Interleukin 1 beta, also known as leukocytic pyrogen, leukocytic endogenous mediator, mononuclear cell factor, or lymphocyte activating factor (IL1-β). Acute aerobic exercises (running, cycling) have been shown to increase monocyte number in young subjects (85, 86) as well as in the elderly (87). In conclusion, there are numerous studies that have examined the acute effects of exercise on monocytes. Interestingly, it seems that brief exercise alters significantly a number of microRNAs (miRNAs) that putatively influence monocytes involvement in vascular health, leading to a novel genomic profile of circulating monocytes, which promotes cardiovascular health. As such, this suggests that, despite the overall stress response and the pro-inflammatory profile, acute exercise exerts a positive effect.

**Macrophages** play a key role in inflammatory responses and are specialized cells involved in the detection, phagocytosis, and destruction of bacteria and other pathogens, as well as in tissue homeostasis and development (88). They can be subdivided into two populations: namely, the pro-inflammatory, anti-tumorigenic M1 macrophages and the anti-inflammatory, pro-tumorigenic M2 macrophages (89). Many studies demonstrate that aging does not change number of macrophages, but it does alter their properties and function (88, 90). For instance, it seems that macrophages of the adipose tissue and the liver of the elderly exhibit a more M1 phenotype when compared to younger subjects (89). Macrophages also play an important anti-inflammatory role and can decrease immune reactions through release of cytokines: briefly, aging is characterized by “dysregulated macrophage-mediated immunosuppression” (89). A recent study demonstrated that during rest, there was an increase in IL-1 and IL-10 produced by macrophages, yet an unaltered number and function of these cells immediately post-exercise (91).

Like macrophages, **neutrophils** play an important role in the innate immune system and are the most abundant granulocyte (40% to 75% of WBCs). Neutrophils have many ways of neutralizing microorganisms: namely, chemotaxis, phagocytosis (engulfment of bacteria, other pathogens or tissue fragments), degranulation of cytoplasmic granules, activation of the respiratory burst, and neutrophil extracellular traps (92). It has been revealed that acute exercise has a profound impact on neutrophil count, potentially mediated by the activation of cathecolamines as well as of growth hormones and cortisol (42, 52). For example, Cannon et al. (58) showed that running at 75% of maximum heart rate (HR_max_) for 15 min increased neutrophil number (from 3.66 to 3.84 cells/mm^3^) in older adults (aged from 61 to 72 years). Cannon et al. (60) in a group of 12 old subjects aged 61–72 years vs. a sample of 9 participants aged 20–32 years, eccentric exercise (45 min at 78% of maximum heart rate) led to the increase of circulating neutrophils.

According to numerous studies, neutrophils recruited into circulation during exercise may be responsible for controlling the elevated levels of oxidative stress in plasma after exercise (93). The lower adhesion of neutrophils and platelets elicited by regular exercise could be an important factor in the prevention of vascular and inflammatory diseases, among others (94).

Furthermore, habitual physical activity is associated with the maintenance of neutrophil migratory dynamics in a sample of 211 healthy older adults aged 67 ± 5 years when compared to 10 young participants (aged 23 ± 4 years). There was no difference in expression of the chemokine receptors CXCR1 or CXCR2 (73).

Few studies have investigated the effect of acute exercise on **leukocytes** in the elderly. For instance, Ludlow et al. (68) found in a sample of 69 subjects aged 50–70 years that the leukocyte telomere length was increased in subjects undergoing moderate physical activity (50-70% VO_2max_), when compared to groups practicing low and high levels of exercise and training. Specifically, the second exercise energy expenditure (EEE) quartile had longer telomere lengths with respect to the first and fourth quartiles but not to the third one, whereas the telomerase activity did not differ among the groups, remaining preserved and stable.

Several studies have demonstrated that acute exercise induces recruitment of **lymphocyte** subpopulations from marginated leukocyte pools from organs into general circulation. Two of these types of lymphocytes are critical for specific immune responses; B lymphocytes (B cells) and T lymphocytes (T cells), for humoral and cellular responses, respectively. Both B and T cells are notably impacted, with elevated serum levels post exercise. Mobilization of these cells is due to redistribution of activated cells with elevated replicative history rather than cells isolated from blood at rest such as CD4+ T cells, CD8+ T cells, CD16+ NK, and CD56+ NK cells (95).

Lymphocytosis is known to occur during exercise or immediately thereafter during the early stages of the recovery phase and is proportional to the intensity and the duration of acute exercise, with an increase in the number of T lymphocytes (and to a lesser extent B lymphocytes) compared to the values measured before exercise (96, 97). Subsequently, during the 24 h following the effort, data reported the same values at rest. Mobilization of these subgroups of cells (T and B) is largely influenced by the action of catecholamines which is the response of the nervous system to energy demand (98). After intense long duration exercise, lymphopenia is reported, with the lymphocyte number decreasing rapidly after exercise. However this phenomenon is transient and, rather than being a real reduction, ig is secondary to lymphocyte redeployment to peripheral side (51).

Mooren et al. (99), reported a continuous exhaustive at 80% VO_2max_ (progressive exercise test on a treadmill ergometer) induces apoptosis in peripheral blood lymphocytes in young (20–30 years) while the moderate intensity exercise performed at 60% of VO_2max_ did not. As indicated in the last study, the subjects were young but there are no studies that have investigated apoptosis in elderly/older subjects depending on exercise intensity which make it difficult to really understand the responses of each type of exercise on this population.

In a sample of 14 young (aged ~22 years) and 33 older adults (aged ~ 65 years) acute maximal exercise induces leukocytosis, mainly due to lymphocytosis and neutrophilia (100–102). In particular, an increase both in CD8+ lymphocytes (153 and 112% respectively) and in CD4+ lymphocytes (57 and 22% respectively) could be detected (63). Specifically, older subjects exhibited higher percentages of memory CD45RO+ CD4+ cells and CD8+ cells pre-exercise and lower percentages of naive CD45RA+ CD4+ and CD8+ cells pre-exercise when compared to younger individuals, even though both groups recruited equal number of naïve and memory cells in response to exercise (103). Despite the higher number of CD3+ cells, the lymphoproliferative response was lower in the elderly subjects. It can be concluded that exercise-induced leukocytosis can occur both in young and old individuals, even though this is less frequent among the elderly, whereas the lympho-proliferative response was different (101, 102).

In addition, Mazzeo et al. (61) examined the changes induced by an acute 20-min bout of aerobic exercise (cycling at 50% of peak power) on lymphocytes in older adults (aged ~69 years) and observed an increase in CD4+ and CD8+ T-cells post-exercise. Thus, during exercise, CD4+ T-cells, CD8+ T-cells, CD19 B-cells, CD16 NK cells, and CD56 NK cells increase in number after intense exercise, whilst they decrease 1-h post-exercise.

Silva et al. (74) recruited a sample of 46 subjects aged 65–85 years, subdivided into three groups (untrained subjects vs. moderately and intensely trained individuals). A significant redeployment of T-cells could be noticed: namely, a decrease in CD45RA+ C-C chemokine receptor type 7 (CCR7)- CD4+ and CD8+ T-cells, an increase in central memory CD4+ T-cells and in effector memory CD8+ T-cells, whilst the number of CD28- T-cells remained unvaried. Furthermore, telomere length in CD4+ and CD8+ T-cells correlated with training intensity.

Minuzzi et al. (75) investigated a sample of 19 male old subjects (aged ~54 years) undergoing the cycle ergometer test and found that acute exercise can lead to a decrease in the percentage of both CD4+ and CD8+ T-cells. Specifically, this effect was due to an increase in short-lived effector cells (SLECs) and to a decrease of senescent naïve, central memory, and effector memory cells. A similar redeployment of CD8+ T cell subsets, induced by acute exercise, was described by Bruunsgaard et al. (62) in a sample of 10 individuals (aged 76–80 years) undergoing a maximal bicycle exercise for 17–20 min, by Spielmann et al. (70) in a group of 102 subjects undergoing the cycling ergometer test, and by Spielmann et al. (71) in a sample of 32 individuals undergoing a 30-min sub-maximal cycling test (at ~80% peak cycling power).

Inconsistent findings were obtained related to cytokines and interleukins (64, 65, 104). After a bout of isokinetic exercise in a sample of 16 subjects (8 participants aged ~67 years vs. 8 individuals aged ~20 years), an increase of monocyte chemoattractant protein-1 (MCP-1, known also as CCL2) (105), IL-6, and IL-8 could be detected whereas the serum levels of IL-4, IL-10, and interleukin-13 (IL-13) remained stable (104). In another study, in a sample of 15 subjects aged 66–78 years, acute eccentric exercise (45 min of downhill running, 16% descent, at 75% VO_2max_) led to increase of IL-1, TNF-α, whereas, IL-6 did not change before and after the exercise (65). Finally, Colbert and colleagues (64) interviewed 3075 subjects aged 70-79 years concerning their previous-week household, walking, exercise, and occupational/volunteer physical activities. Moderate weekly physical activity (defined as, at least 180 min/week of walking, occupational/volunteer physical activities) was found to correlate with lower levels of CRP, IL-6, and TNF-α.

**Natural killer cells** (also known as NK cells, K cells, or killer cells) are lymphocytes that share a common progenitor with all subsets of T and B lymphocytes. NK cells are a class of cytotoxic lymphocytes that control several microbial infections and tumor cells by limiting their spread and removing damaged tissue (106, 107). They express surface markers, CD16 and CD56, but do not express CD3. On the basis of the level of CD56 expression, they can be roughly subdivided into two major subsets: namely, CD56^bright^ and CD56^dim^ cells, which exhibit different phenotypical and functional characteristics. CD56^dim^ represents a mature subset of NK cells, with exclusive migratory potential for non-lymphoid tissue and potent effector capabilities, such as the capacity to produce and release high amounts of perforin and granzyme. CD56^bright^ usually reside in secondary lymphoid organs, and express cell-surface molecules such as the lymphoid tissue homing makers L-selectin (CD62L) and CCR7 (32, 108, 109). CD56^bright^ and CD56^dim^ cells also differ in terms of response to exercise, with CD56^bright^ being less responsive to physical activity compared to CD56^dim^ (110). Whilst interferon alpha (IFN-α) (111) and interleukin-2 (IL-2) (112) increase cytolytic activity of NK cells, immune perturbations, some prostaglandins and infections such as CMV can decrease activity of NK cells (112, 113).

Aging induces changes to the phenotype and function of NK cells. Studies found that the number of NK cells increases in elderly individuals caused by expansion of the CD56^dim^ subset and the accumulation of CD57+ long-lived NK cells (114). CD56^bright^ cells, instead, tend to decrease (114), as well as NK capacity for killing decreases with age (115). Others have shown that low to moderate cycling exercise leads to an increased NK cells cytotoxicity (NKCC) (116). In particular, Targan and collaborators (116) found that in a sample of 10 healthy volunteers (of unreported age and gender composition) bicycle ergometer (pedaling at speeds of approximately 25 m.p.h. for 5 min) leads to the recruitment of some NK subsets: cells which can bind targets but are non-cytotoxic and to the increased capacity of IFN of inducing overall lytic ability.

Generally, most types of exercise can increase NK cell function and number (57, 72, 117, 118). Brahmi and colleagues (117) recruited both trained and sedentary individuals, who underwent a progressive cycle ergometer test using an incremental work load of 15 W (90 kpm), increasing every minute. NK activity against K562 reached maximum levels immediately after exercise, decreased 120 min later, and then slowly came back to pre-exercise levels within 20 h. Fiatarone et al. (57) observed an increase in NK activity and response in a sample of 9 subjects aged 65 years and above (vs. a group of 8 participants aged 21–39 years) undergoing the cycling ergometer test.

A mechanism explaining NK mobilization and redistribution could be that the binding of epinephrine as well as increased serum serotonin releases NK cells from endothelial tissue *via* a decrease in adhesion molecules following acute exercise (119, 120). Shear stress could play a major role too (119).

Among young subjects, Bigley and co-workers (121) had found that three 30-min cycling bouts at −5%, +5%, and +15% of lactate threshold can lead to redeployment of NK cells in a sample of 16 healthy cyclists. This redeployment is stepwise and preferential in the sense that it involves NK-subsets exhibiting a high differentiation phenotype (KIR+/NKG2A– vs. medium-differentiated KIR+/NKG2A+ and low-differentiated KIR–/NKG2A+). Bigley and co-authors (72) were able to replicate this finding in a sample of 40 subjects (50–64 vs. 23–39 years) undergoing 30 min of cycling exercise at 80% of maximum power. Interestingly, the post-exercise response to CMV was similar between young and old individuals (72).

Summarizing, acute exercise in older subjects increases NK cell both in terms of number and activity/function, mobilizing and redistributing them. Furthermore, exercise increases circulating numbers of neutrophils; potentially increases secondary antibody response to booster injections; changes circulating T-cell populations (decreasing naïve CD8+ T-cells, increasing SLEC CD8+ T-cells), induces leukocytosis; and modulates T-cell proliferation (95). Inconsistent findings related to cytokines and interleukins are, instead, reported in the extant scholarly literature.

However, as can be seen from Table 2, most studies did not analyze the effect of gender on the changes in NK cell properties during exercise. This is particularly important in younger women due to the known impact of the cycle of immune parameters, and could also be a mediating factor for the acute effects of exercise in older women (122, 123). Another limitation that should be emphasized is that studies focused on older subjects but not on oldest individuals (aged >80 years old). Taking into account the global phenomenon of aging and the lengthening of the human life span, this is an important gap in our knowledge that should be properly addressed by future research. Furthermore, the studies summarized so far did not assess CMV serostatus, so the results are difficult to interpret in an unambiguous way (32).

## Impact of chronic exercise on immune cells

In general, studies on the effect of chronic exercise on immune cells and immunity function have been focused mostly on healthy young people, athletes, elderly, oncologic or HIV patients, with the overall goals of ascertaining the extent of immune decline in athletes due to extreme exercise training (overtraining/ excessive training) or finding the responsible factor that help to improve immunity responses in the elderly or the immunocompromised without clear evidence of the impact of exercise training type itself. Long-term effect on immune function in the elderly is less debated. In their systematic review, Cao Dinh et al. (22) reported that most studies were conducted in young (~20–40 years) and middle-aged (~40–50 years) and documented an increase in NK cells activity and T lymphocytes occurring without apoptosis.

The intriguing observation is that (1) most studies debated the effect of aerobic and resistance training, while no evidence on the effect of anaerobic training such as sprint training on resting or in response to acute exercise were found in the elderly population (>60 years) and (2) the exercise induced apoptosis in senescent cells in the elderly is still not debated.

On the other hand, it is noteworthy that intensity of training required to achieve a certain goal differs according to age groups [American College of Sports Medicine (ACSM)]. Thus, for better interpretation of results it is essential to consider that older subjects (> 60 years) have low fitness levels compared with more young (20–40 years), and they can only reach the significant exercise training effect with a training heart rate as low as 40–50% of heart rate compared with a person with high fitness levels. Therefore, these methodological considerations indicate that the findings of studies need to be interpreted with caution.

## Endurance training on immune cells

A healthy amount of regular exercise provides an overall benefit to the immune system. However, more than one component of the immune system may be weakened by excessive training (124). In fact, long-term intensive training may result in a decline in function of innate immune cells' capacity to respond to acute challenges, contributing to an elevated risk of infection (125). Hence, immune response depends on training intensity and duration (45, 48, 126). Despite the immune system's vulnerability to prodigious exercise training, the overall anti-inflammatory effect of exercise may reduce the risk of age-related chronic disease characterized by chronic low-grade inflammation (e.g., cancer, type 2 diabetes, heart, and Alzheimer's disease) (127).

**Dendritic cells** differentiate from monocytes to become professional antigen presenting cells (APCs). They form a part of the dendritic cell/macrophage continuum that presents antigen material on their MHC I/II to both T CD8+ and CD4+ cells, respectively, and play an important role as a messenger between innate and adaptive immune system. Two subsets of DCs are recognized: namely, DCs of myeloid origin (including the conventional DCs in the blood, interstitial DCs in tissues, Langerhans cells in the skin and monocyte-derived DCs) and the DCs of lymphoid origin (or plasmacytoid DCs) (128). Aging does not seem to affect the overall number of DCs, even though it could lead to decrease in the number of Langerhans cells in the skin and of plasmacytoid DCs (128). Della Bella and co-authors (129) showed that in a sample of 70 healthy subjects aged 20–92 years the number of myeloid DCs progressively declines with age, together with a decrease of CD34+ precursors and an increase of circulating monocytes, suggesting that the entire differentiation process of antigen presenting cells (APCs) is partially dysregulated in the elderly. Furthermore, DCs from aged individuals appeared to have a more mature phenotype and an impaired ability to produce and release IL-12 upon stimulation.

Recent data suggests that Tai Chi Chuan elevates the number of circulating myeloid DCs but not the plasmacytoid DCs in middle-aged individuals ~53 years (130). However, few studies have examined the effects of endurance training on stem cells number and function.

Concerning stem cells, Thijssen et al. (131) found that endurance training induced significant increases in numbers of hematopoietic stem cells (HSCs) and endothelial progenitor cells (EPCs) in older subjects.

Other studies have shown that moderate exercise increases **neutrophil** function (chemotaxis, phagocytosis, and oxidative burst activity). In contrast, severe or heavy exercise diminished these cell activities without affecting chemotaxis and degranulation (92, 132).

In older individuals, some studies that have explored the effect of endurance training on immunity response, have found no changes in circulating neutrophil count (103, 133). Yan et al. (134) found that neutrophil phagocytic ability in older adults is attenuated by moderate exercise compared to a control group. Given that few studies have investigated the impact of endurance training on neutrophils; it is difficult to make conclusive statements regards the role of endurance training on neutrophils in the elderly.

**Eosinophils** are a type of white blood cells that defend against parasites and infectious agents. Aging induces several changes in eosinophils such as decreases in blood eosinophils number in the elderly. However, there is no alteration to the function of these cells in young and older group with asthma (135).

**Basophils** are a type of leukocyte which represent 1% of circulating WBC. These cells play a key role in the inflammatory response. The effect of endurance training on eosinophils and basophils is rather unexplored but endurance training has been found to induce no change in eosinophils and basophils levels (122).

Shimizu et al. (136) reported that 6 months of moderate exercise training performed by elderly individuals (61–79 years) led to increased expression of CD28 on CD4+ T cells. Moreover, Nieman et al. (48) compared the immune response before and after training and they found that 12 weeks of training did not induce changes in T cell proliferation in older women (65–85 years). In contrast, Woods et al. (103) examined the effects after 6 months aerobic exercise on T cell compartment in older adult participants. They found that T cell proliferation increased in the experimental group compared to a control group and was responsive to mitogenic stimulation. Few studies indicate that endurance training has no effect on T cell function and cell frequencies in the elderly population (62). Future studies may wish to elucidate why endurance training fails to improve T cell function and determine whether there is a genetic predisposition, controlling the inter-individual variation in responses to immune function after different training regimes.

Kapasi et al. (137) found that 32 weeks' moderate aerobic training had no effect on T-cell proliferative responses and the soluble production of cytokine activity in frail elderly nursing home residents. Fairey et al. (138) showed that aerobic exercise training increased T cell proliferation (by 218 per dpm × 10^6^ cells) in post-menopausal breast cancer survivors, whereas . van der Geest et al. (139) found that in a sample of people aged 80 years, walking 30 km per day lead to an increase of CD4+ T-cells.

Nieman et al. (48) has shown a higher **NK cell** activity in older woman (67-85 years) after walking for 12 weeks compared to a callisthenic control group. Moreover, it has also been shown that 6 months of moderate aerobic training practiced by older adult men aged 65 years, increased NK cell cytolysis (103). Recently Della Bella et al. (134), showed that concentration of NK cells (CD16+ and CD56+) increased in the older adults (>60 year) who performed regular moderate exercise for 1 h twice per week.

Distinct types of exercise and prescriptions (frequency, duration, and intensity) and sex discrepancies could be responsible for these discordant results Table 3. Also, the changes of composition of NK cells caused by aging could be responsible for discrepancies amongst results, such as CD57, cluster of differentiation 158 (CD158), killer lectin-like receptor 61 (KLR61) (144) and cluster of differentiation 94 (CD94) receptors (145). NK tumor cytoxicity increased following a 16-week exercise intervention in 14 elderly women, due to increased number of NK cells (56).

**Table 3:** The effects of endurance training on the immune system.

**Reference**	**Subjects age gender**	**Type of acute exercise**	**Results**
Nieman et al. (48)	Sex: female Age: 65–85 Number: 42 (12 trained/30 untrained)	Endurance competition (race)	↑NK cell activity ↓incidence of URTI ↑T cell proliferation
Woods et al. (103)	Sex: male/female Age: 65 Number:29 (14 trained/15 untrained)	Aerobic exercise: 10–40 min/session 50–65% $\dot{V}$O_2max_3 times/week 6 months	↑T cell proliferation ↑NK cell cytotoxicity ↔ Neutrophil, lymphocyte (TCD4/ TCD8), monocyte counts ↓memory(CD45RO+) CD4+T Cell counts, CD4:CD8 T-cell ratio
Ogawa et al. (140)	Sex: female Age: 63 Number: untrained 12 Trained 9	Regular exercise Training: walking $\dot{V}$O_2peak_: 32.2 (ml^.^kg^−1.^min^−1^)	↑ CD8+/IL2 ↑CD4/IFN?
Fairey et al. (138)	Sex: female Age: 59 Number: 53 untrained/28 trained 25	Cycle ergometer 3 times/week 15 weeks	↑NK cell cytotoxicity ↔ Unstimulated 3H thymidine uptake by peripheral blood lymphocyte
Shinkai et al. (141)	Sex: male Age: 63–65 Number:17 older adult runners/19 controls	Endurance training: running	↔ NK cell activity ↑ IL2, IFN?, IL4 ↔ circulating counts of immunocompetent cells ↑ T cell proliferation
Kapasi et al. (137)	Sex: female Age: 87 Number: 96 untrained and 94 trained	Endurance and resistance exercise training 5 days/week 8 months	↔ Lymphocytes subpopulations or Activation markers (CD28, CD25, HLA-DR) ↔NeotropinsTNF-RII production in serum
Drela et al. (142)	Sex: female Age: 62–86 Number: 30	2 year physical activity program	↑ lymphocytes expressing intracellular IL2 ↔ Intracellular expression of IL4 and IFN?
Shimizu et al. (136)	Sex: female/male Age: 61–79 Number:48 untrained 20 Trained 28	Exercise training session 5 days/week 6 months	↔Leukocytes, Lymphocytes CD3 T cells ↑CD4+ CD28+ cells
Thijssen et al. (131)	Sex: male Age: 19–28 67–76 Number: 16	Endurance training 8 weeks	↑ HSCs and EPCs in older subjects
Yan et al. (134)	Sex: male Age: young (20–39) Middle aged (40–59) Elderly (more than 60 years) Number: elderly (20 untrained/ 28: trained)	Moderate exercise training	Trained older adult group: ↑phagocytic capacity of neutrophils ↑NK cells CD16+ CD56+
Thijssen et al. (131)	Sex: male Age: 71–73 Number: 14 (7 untrained and 7 Trained)	8 weeks of endurance training	↑ circulating HSCs and EPCs
Kohut et al. (143)	Age: 64 Number: 14 trained 13 untrained	Endurance training 65–75% heart rate reserve (HRR), 25–30 min, 3 days per week, for 10 months	↑ antibody to influenza immunization

*↑, greater response;↓, lower response; ↔, No difference due to exercise training; CD, cluster of differentiation; URTI, upper respiratory tract infection; CRP, C-reactive protein; IL, interleukin; IFN, interferon; ${\dot{V}O}_{2peak}$, peak oxygen uptake; maximal; NK, natural killer*.

## Effect of resistance training on immune cells

Currently, ambiguity remains over the influence of resistance training on immunity in older adults (Table 4) (154). Some studies (147–150, 155) found that short-term resistance training intervention (8–12 weeks) in the healthy elderly (between 65–84 years of age) had no beneficial or detrimental effect on (lymphocytic counts, apoptosis, proliferation…etc), while other studies have found beneficial effects on inflammatory status in older subjects with breast cancer or obesity syndrome (151, 152, 156) or healthy older ones (69 years) (153), and the combination of resistance and aerobic or endurance training reported a concrete change in immune cells levels in the elderly. For example, Timmerman et al. (81) reported that 12 weeks of aerobic and resistance exercise training lowered (CD14+, CD16+) **monocyte** frequencies in blood of older adults, while no changes were observed in toll-like receptor 4 (TLR4) expression, a type of protein playing an important role in innate immunity. TLR4 is expressed on sentinel cells and recognizes molecules derived from microbes. McFarlin et al. (157) found that TLR4 expression is lower in resistance trained women (65–80 years) compared to untrained peers. Up-regulated TLR activation is implicated in the manifestation of hypertension and chronic low-grade inflammation (158, 159). Stewart et al. (66) showed also that 12 weeks of resistance exercise (50% 1RM) decreased the TLR4 expression on monocytes in elderly subjects.

**Table 4:** The effects of resistance training on the immune system.

**Reference**	**Subjects' age and sex**	**Type of acute exercise**	**Results**
Timmeman et al. (81)	Sex: female/male Age: 65–80 years Number:30 15 untrained and 15 trained	RT	↓percentage of CD14+CD16+, monocytes and CRP
Cambell et al. (146)	Sex: female Age: 50–75 years Number: 173 (86 untrained and trained87)	12 months of progressive aerobic and resistance training 40–75%: heart rate 45 min/session 5 times/week	↔ NK cell cytotoxicity ↔ CD4+/CD8+ ratio ↔ T-cell proliferation ↔ Lymphocyte T cell CD4 Lymphocyte T cell CD8 NK cell, B cell
Flynn et al. (147)	Sex: female Age: 67–84 years Number:29 (15 trained and 14 untrained)	10 weeks of resistance training	↔ lymphocyte proliferation ↔ NK cell cytotoxicity
Kapasi et al. (137)	Sex: female Age: 87 years Number: 190 (96 untrained and 94 trained)	Endurance and resistance exercise training 5 days/week 8 months	↔ Lymphocytes subpopulations or Activation markers (CD28, CD25, HLA-DR) ↔Neotropins TNF-RII production in serum
Raso et al. (148)	Sex: female Age: 60–77 years Number: 42	12 months of moderate RT	↔NK cells cytotoxicity ↔lymphocyte proliferation ↔CD4+, CD8+ T cells expressing CD28+
Bobeuf et al. (149)	Sex: male and female Age: 66.7 ± 3.7 years (61-73 years) Number: 29 (16 cases, 13 controls)	3 days per week, 3 sets of 8 repetitions at 80% 1-RM	↔red blood cells ↔hemoglobin ↔hematocrit ↔platelets ↔leukocytes ↔neutrophils ↔lymphocytes ↔monocytes ↔mean corpuscular volume ↔mean corpuscular hemoglobin ↔mean corpuscular hemoglobin concentration ↔red cell distribution width
Rall et al. (150)	Sex: NR Age: 65–80 years Number: 6	12 week of progressive resistance strength training	↔lymphocyte proliferation
Serra et al. (151)	Sex: female with breast cancer Age: range 48–75 years Number: 11	16 weeks of RT: progressive contraction of major muscle groups leg and chest press, knee extension, leg curl, row, abdominal crunch, and bicep cur	changes in inflammation with ↓ pro-inflammatory cytokines TNF-a, IL-6sR, and SAA in both plasma and adipose tissue and increases in the pro-proliferative IL-8
Tomeleri et al. (152)	Sex: obese women Age: range 40-68 years Number: 38	Resistance training: 8 whole-body exercises for 3 sets of 10–15 repetition maximum (RM) carried out 3 times a week	↓ pro-inflammatory biomarkers levels such as IL-6, TNF-α, and CRP
Mejías-Peña et al. (153)	Sex: female and male Age: 69.6 years Number:26 healthy	8-week resistance training	Stimulates autophagy, Prevents NLRP3 inflammasome activation, ↓apoptosis in PBMC

↑, greater response;↓, lower response; ↔, No difference due to exercise training; CD, cluster of differentiation; URTI, upper respiratory tract infection; CRP, C-reactive protein; IL, interleukin; IFN, interferon; $\dot{V}$O_2peak_, peak oxygen uptake; maximal;$\dot{\;V}$O_2max_, maximum oxygen uptake;NK, natural killer; PBMCs, peripheral blood mononuclear cells; NR, not reported

For **Neutrophils**, only a few studies investigated the effect of resistance exercises on their circulating levels. Recently, Bartholomeu-Neto et al. (156) explored the effect of resistance training (moderate-intensity physical training (70% of 1RM) with session including nine exercises: horizontal leg press, knee extension, knee flexion, bench press, triceps extension in the pulley, biceps curling, seated rowing, plantar flexion, and abdominals. They found higher phagocytic activity in healthy older women. This phagocytic activity is determined by the phagocytosis index of neutrophils (but not of monocytes). In contrast, the training did not significantly influence the oxidative activity of either neutrophils or monocytes in elderly women.

Kapasi et al. (137) found no change in activation markers (CD28, CD45RA, CD45RO, or HLA.DR) and subpopulation **lymphocytes** (CD4+ or CD8+ T cells) in older adult nursing home residents who performed 32-weeks of combined endurance and resistance exercise. Similarly, Flynn et al. (147) reported 10 weeks' resistance training did not alter T cell proliferation in older women (67–84 years). Moreover, recent studies (146, 148) showed that 12 months' moderate resistance training failed to induce changes in T cell proliferation and NK cells in older women. Raso et al. (148) found that a 12-month, moderate resistance training program produces significant improvement in muscle strength of healthy, elderly women without significant differences between the two groups at any time point for total lymphocyte number or its subsets (CD3+, CD4+, CD8+, CD19+, and CD56+ cells). In addition, Rall et al. (150) found that 12 weeks' progressive resistance training practiced by elderly volunteers (aged 65–80 years) did not affect lymphocyte proliferation. The inconsistent results could be due to different doses of mitogen, types of exercise (running, cycling, walking), exercise prescription and the duration of proliferation (95).

Campbell et al. (146) found that 12 months' progressive aerobic and resistance training practiced by postmenopausal women (50-75 years) has no effect on **NK cell cytotoxicity**. Bermon et al. (155) investigated the effect of 8-week strength training in elderly individuals (aged ~70 years) on NK cells and found that resistance training did not induce changes in NK cell function. Bermon et al. (160) found that n sedentary older adults, unlike young subjects, strength exercises can induce a transient decrease in NK cell count which can be canceled by a short-term strength conditioning. However, Fairey et al. (138) found that 10 weeks' resistance training improved NK cell function in post-menopausal breast cancer survivors aged (65–85 years).

## Effect of sprint training on immune cells

Sprint training (ST) is considered as repeated bouts of exercise at supramaximal intensity. Sprint training is time efficient method of improving performance (161), and cardio-metabolic health (162). Moreover, it induces alterations in immunity such depression of neutrophil function and an increase of cytokines. However, immune responses depend on exercise training duration, intensity, and subject population.

Regarding inflammatory markers, inconsistent findings have been reported in the literature. Allen and colleagues (163) performed a randomized controlled trial, randomly allocating 55 sedentary adults (aged 49.2 ± 6.1 years). Twenty were randomized into high intensity interval training, whereas 21 and 14 to prolonged intermittent ST (PIST) and to a sedentary control group, respectively. HIIT and PIST groups performed three training sessions per week for 9 weeks. On a cycle ergometer, at the end of the trial, markers of systemic inflammation (CRP and TNF-α) remained unchanged across all groups. However, Harnish and Sabo (164) reported increased levels of IL-6, IL-10, and TNF-α after SIT in a sample of 13 men and two women aged 23.8 ± 3.5 years.

The influence of ST on **neutrophils** has been described in few studies, yet a single session of ST typically causes a decrease in neutrophil function (oxidative burst and degranulation). However, the depression of neutrophils is caused by oxidative stress and stress hormones (165).

The effect of ST on **lymphocytes and lymphocyte proliferation** has been investigated in rats whereby ST during for 5 weeks increased FeG+ lymphocyte number (166). Although, the effect of ST on lymphocytes in the elderly is rarely investigated, a recent study has shown in 53 sprinters (22.2 ± 1.1 years) that any supplementary increase in lactic acid level does not cause an additional increase in lymphocyte subsets after four sprint intervals (1,000 meters at 85% of a subject's maximal velocity) (167). In addition, there are studies concerning the effect of anaerobic exercise in general, such as the study of Hack et al. (168) that investigated the influence of 8-week anaerobic exercise training program in healthy untrained subjects. They indicate impairment of the number and activity of CD4+ T cells, which might be linked to metabolic factors such as glutamine.

On other hand, there is limited information concerning the influence of ST on **NK cells**. Marshall-Gradisnik et al (169) performed a 10-s all-out cycle sprint test in healthy young males at week 0 and at week 6 and found a significant increase in NK cytotoxic activity (NKCA), whilst the NK number did not significantly increase. Another recent study found that the recruitment of NK cells depends on the levels of lactic acid, higher levels of lactate increase NK cell activity and number after four sprint intervals accumulating to 1,000 meters at 85% of a subject's maximal velocity in 53 sprinters (aged 22.2 ± 1.1 years) (167).

## Conclusion

The present review has synthesized and discussed the current evidence for the role of exercise interventions in influencing lymphocytes activity in older subjects. The most marked changes occur in NK cells and the general proliferation of immune cells (i.e., the cell proliferative capability and involution of tissues and organs). This is important because advanced age induces decreased proliferative response.

Many studies show the positive effect of exercise on the immune system such as elevation in T-cell proliferative capacity, increased neutrophil function, and NK cell cytotoxic activity.

The magnitude of exercise-induced immune changes in older adults were different between studies, possibly due to the different protocols, methodologies, ages, sexes, and testing procedures.

Future detailed studies should examine the impact of exercise on immune cell function, such as neutrophils and elucidate the underlying mechanisms responsible for the alterations and the subsequent effect on immunity and health. It will also be critical to elucidate the inter-individual responses in changes to immune parameters (e.g., T-cell activity) after different forms of exercise training, which could account for some of the findings amongst the current literature.

Ultimately, these studies will contribute to our understanding of the role exercise training may have in preventing immunosenescence and disease, or improving health span.

## Author contributions

MS designed and conceived the study. MS and NB drafted the study. MS, MG, JD, LH, DS, JP, and NB critically revised the manuscript.

### Conflict of interest statement

The authors declare that the research was conducted in the absence of any commercial or financial relationships that could be construed as a potential conflict of interest.

## Abbreviations

ACSM: American College of Sports Medicine
APC: antigen presenting cell
CCR7: C-C chemokine receptor type 7
CD4: cluster of differentiation 4
CD8: cluster of differentiation 8
CD11b: cluster of differentiation 11b
CD18: cluster of differentiation 18
CD19: cluster of differentiation 19
CD28: cluster of differentiation 28
CD45RA: cluster of differentiation 45RA
CD45RO: cluster of differentiation 45RO
CD56: cluster of differentiation 56
CD62L: cluster of differentiation 62 ligand
CD94: cluster of differentiation 94
CD158: cluster of differentiation 158
CK: creatine kinase
CMV: cytomegalovirus
con-A: concanavalin A
CRP: C-reactive protein
CXCR1: C-X-C chemokine receptor type 1
CXCR2: C-X-C chemokine receptor type 2
DC: dendritic cell
EEE: exercise energy expenditure
EPC: endothelial progenitor cells
HLA-DR: Human Leukocyte Antigen – antigen D Related
HR: heart rate
HR_max_: maximum heart rate
HRR: heart rate reserve
HSC: hematopoietic stem cells
hsCRP: highly sensitive C-reactive protein
hTERT: human telomerase reverse transcriptase
IFN: interferon
IFN-γ: interferon-gamma
IL-1: interleukin 1
IL1-β: interleukin 1-beta
IL-2: interleukin 2
IL-4: interleukin 4
IL-6: interleukin 6
IL-12: interleukin 12
IL-13: interleukin 13
KLR: killer lectin-like receptor
KLR61: killer lectin-like receptor 61
miRNA: microRNA
mRNA: RNA messenger
NK: natural killer
NKCA: natural killer cytotoxic activity
NKCC: natural killer cellular citotoxicity
NR: not reported
PBMC: peripheral blood mononuclear cell
PHA: phitoemagglutinin
PIST: prolonged intermittent sprinting training
PPD: purified protein derivative
PWM: poke weed mitogen
RM: repetition maximum
RT: resistance training
SLEC: short-lived effector cells
ST: sprint training
TGF: transforming growth factor
TGF-β1: transforming growth factor beta 1
TLR2: Toll like receptor 2
TLR4: Toll like receptor 4
TNF-α: tumor necrosis factor alpha
URTI: upper respiratory tract infection
WBC: white blood cell
WHO: World Health Organization.
